# The potential impact of allied health professional telehealth consultations on health inequities and the burden of treatment

**DOI:** 10.1186/s12939-022-01689-2

**Published:** 2022-06-30

**Authors:** Nicola Eddison, Enza Leone, Aoife Healy, Carolyn Royse, Nachiappan Chockalingam

**Affiliations:** 1 Centre for Biomechanics and Rehabilitation Technologies, Staffordshire University, Technologies Leek Road, ST4 2DF Stoke on Trent, UK; 2 Royal Wolverhampton NHS Trust, WV10 0QP Wolverhampton, UK; 3 Dorset County Hospital NHS Foundation Trust, DT1 2JY Dorchester, Dorset, UK

**Keywords:** Telehealth, Allied Health Professionals, Health inequities

## Abstract

**Background:**

The COVID-19 pandemic resulted in a rapid shift to remote consultations. The study aimed to explore the prevalence of telehealth consultations amongst allied health professional (AHP) services in the UK National Health Service (NHS), and the potential impact on health inequities and burden of treatment for patients.

**Methods:**

Cross-sectional online survey. Participants were practising UK registered AHP and/or AHP service manager in an NHS/social care/local authority service. Data was collected between May – June 2021.

**Results:**

658 participants took part in this study, including 119 AHP service managers, managing a total of 168 AHP services, and 539 clinicians. 87.4% of clinicians and 89.4% of services represented were using telehealth consultations as a method of delivering healthcare, the majority reported their services were planning to continue using telehealth post COVID-19 restrictions. Participants reported a lack of technological skills for patients as the most prevalent barrier affecting the patient’s ability to conduct a telehealth consultation, followed by a lack of technology for patients. These were also reported as the biggest disadvantages of telehealth for patients. The majority of clinicians reported a reduction in the cost of parking/transport to attend hospital appointments as a patient benefit of telehealth consultations. Reported benefits for clinicians included saving travel time/costs and allowing flexible working, while benefits to the AHP service included patient flexibility in how their appointments are conducted and reducing the potential exposure of staff to communicable diseases.

**Conclusions:**

The current large-scale implementation of telehealth in NHS AHP services may increase disparities in health care access for vulnerable populations with limited digital literacy or access. Consequently, there is a danger that telehealth will be considered inappropriate and thus, underutilised, negating the potential benefits of sustainability, patient empowerment and the reduction in the burden of treatment.

**Supplementary Information:**

The online version contains supplementary material available at 10.1186/s12939-022-01689-2.

## Background

As a result of the Covid-19 pandemic the United Kingdom’s (UK) National Health Service (NHS) rapidly introduced changes to out-patient services, consequently, access to face-to-face out-patient appointments became a ubiquitous barrier. The task of rapidly re-designing how allied health professional (AHP) services continued to deliver care by adapting to the restrictions imposed by the pandemic was significant, as AHPs [1] are the third largest workforce in the NHS with over four million patient contacts per week [2]. It resulted in the rapid expansion of telehealth as an alternative medium of delivering healthcare.

The use of telehealth is not new, it has been estimated that more than 60% of all health care institutions and up to 50% of all hospitals in the United States currently use some form of telehealth [3]. Although previously less prevalent in other countries [4, 5], the unprecedented events of the COVID-19 pandemic have necessitated a rapid expansion of its use in NHS services [6–9]. This rapid introduction of telehealth has caused concern regarding equity of access [10], reporting that socioeconomic factors significantly affect patient access to telehealth [10], due to the cost of internet access, and other information and communication technology (ICT). With internet and other ICT utilisation declining significantly with increasing age beyond 60 years, this may also create a barrier in accessing healthcare within this age group [11]. Patients with hearing and/or vision impairment, cognitive impairment and having a lack of suitable space to carry out a telehealth consultation may also experience inequities of care when accessing telehealth [12].

The potential inequity of access to healthcare via telehealth amongst populations may lead to widening health inequities. Dahlgren and Whitehead [13] examined the concept of ‘equity' in health and discussed the considerable health inequities between socio-economic and occupational groups in Europe. Outlining a ‘rainbow’ of wider determinants of health, [14] including concepts such as 1) general socioeconomic, cultural, and environmental conditions; 2) living and working conditions; 3) social and community networks; 4) lifestyle factors; and 5) individual factors such as age, sex, and constitution.

The nature and complexity of health inequities make them challenging to tackle. In 2008 the UK government, concerned about widening health inequities, commissioned a review chaired by Professor Sir Michael Marmot [15]. The report recommended that reducing health inequities would require action and specific policy objectives. The report was reviewed 10 years later [16] and far from health inequities reducing in this time; by the measures used within the report, health and health inequity had generally worsened (e.g., people can expect to spend more of their lives in poor health). This lack of equality matters and drastically affected outcomes during the COVID-19 pandemic, with unequal burdens carried by different population groups and regions in the UK [17–19].

Digital expansion is cited as a key priority of the NHS Long Term Plan with the aim of reducing face-to-face outpatient appointments by up to a third [20]. However, the rapid digitalisation of NHS services during the pandemic with the introduction of digital technology and innovation to provide telehealth consultations, education, condition management and help and advice to patients, has raised concerns amongst the public, media, and scientific literature [21, 22].

Statistics show that 96% of UK homes have internet access [23], and 95% of people in the UK have a mobile phone [24] of which 88% are smartphones [25]. However, whilst data shows the percentage of adults who have used the internet in 2020 was as high as 92.1% in the UK, this drops to 81.4% of people who consider themselves to have a disability, 85.5% for those aged 65–74 years of age and 54% of those aged over 75 years [26]. One must also distinguish between owning data-enabled devices and having the digital skills and financial resources to use them to conduct telehealth consultations [27]. It is estimated that the number of people in the UK lacking basic digital skills is declining, even so, in 2018, 8% of people in the UK (4.3 million people) were estimated to have zero basic digital skills. A further 12% (6.4 million adults) were estimated to only have limited abilities online [28]. Although digital literacy has been increasing over the last decade in the UK, it is estimated that 7.9 million people will still lack digital skills in 2025 [28].

9.2 million people (14%) in the UK were considered to be living in poverty in 2019/2020 [29]. The rapid digitalisation of NHS out-patient appointments has the potential to introduce or widen an existing inequity for those groups of patients who either do not have the digital literacy to conduct telehealth consultations or cannot afford video-enabled digital equipment, like smartphones or tablets. This is important as video communication is associated with higher patient understanding and satisfaction compared with telephone communication [30, 31].

Telehealth has the potential to both reduce health inequities (e.g., reduction in financial cost to patients of attending hospital appointments) and to increase health inequities due to digital exclusion within certain groups. In the current recovery period of the NHS, telehealth can also limit waiting time inequity of those needing face-to-face appointments for diagnosis or treatment. Thus, it is vital to understand the mechanisms by which this can happen so that services can realise the benefits that digitally-enabled services can offer [32]. The aim of this study, therefore, was to explore the prevalence of telehealth consultations within NHS AHP services and the experiences and opinions of AHP clinicians and service managers using telehealth in an NHS setting.

## Method

 A cross-sectional online survey was distributed via all 14 NHS AHP professional bodies, the AHP federation, England NHS lead networks (via NHS England AHP Regional Leads), the AHP Public Health England Lead networks, The Orthotic and Prosthetic Networks, The National Orthotics Managers’ Association Group, RESTORE network (Sharing Thoughts for Optimising Recovery and Engagement, a network in Dorset, Somerset and South Wiltshire) NHS AHP collaboration platform via the national AHP virtual hub, the Physiotherapy Research Society, all NHS Trusts in the UK and via social media open to all UK AHPs working in the NHS. The survey opened on 7th May 2021 and closed on 13th June 2021. At the time of this survey there were varying COVID-19 restrictions across the UK including a requirement for social distancing, a requirement to work from home where possible and limitations on mixing with friends and family amongst a plethora of other restrictions. Participants in this study were required to be a practising UK registered AHP and/or AHP service manager in an NHS/social care/local authority service. If participants held both a clinical and management role, they were asked to choose whether they wished to respond to the survey as a clinician or as a service manager. If service managers managed multiple AHP services, they were asked to provide responses for up to a maximum of three services.

The survey (see Online Supplementary file 1) was designed to gather information on the use of telehealth in NHS AHP services, focusing on the following areas; (1) prevalence of telehealth consultations in AHP services, (2) barriers to the use of telehealth consultations for patients and clinicians/AHP services, (3) perceived benefits and disadvantages to the use of telehealth consultations for patients and clinicians/AHP services, (4) available telehealth guidance for clinicians, (5) telehealth consultation training for clinicians, (6) funding for telehealth consultations, and (7) effect of telehealth consultations on healthy behaviour conversations. Qualtrics (Qualtrics International, USA) was utilised to administer the survey. This paper will focus on areas 1–3, the other areas will be discussed elsewhere. For the purposes of this project telehealth was defined as a telephone or video/virtual consultation with a patient.

## Results

### Participants

**Fig. 1:**
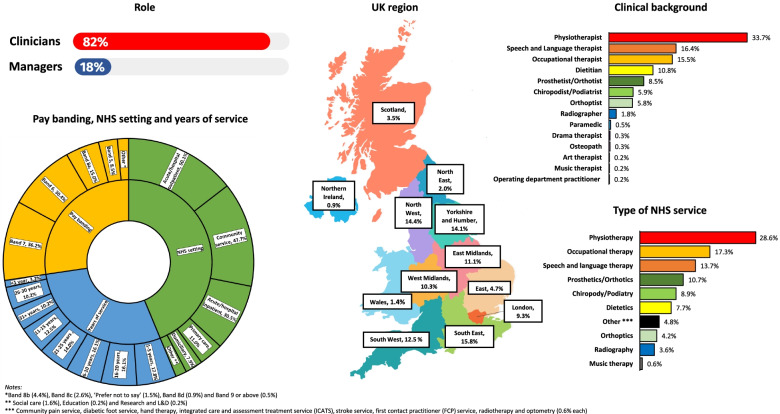
Demographic characteristics of survey participants

658 participants took part in this study, which included 119 AHP managers, managing a total of 168 AHP services, and 539 clinicians. We received AHP representation from each of the 14 NHS AHP professions. Physiotherapists, speech and language therapists, occupational therapists and dieticians accounted for 65.65% of all responses, with physiotherapy, occupational therapy and speech and language therapy making up 100 of the 168 services (59.53%) represented (see Fig. 1). The pay band and thus, skill and experience of the participants in this study ranged from Band 5 to band 9 and above, working across a range of NHS settings delivering patient care in an AHP service. The NHS settings in which most of the respondents worked were acute/hospital outpatient, community service and acute/hospital inpatient, with some participants working in more than one setting.

### Use of telehealth

Telehealth consultations were being used as a method of delivering healthcare by 87.4% (471/539) of clinicians who took part in this study and 89.4% (135/151) of represented services. Most reported that they started using telehealth in 2020, 80.7% (392/486) of clinicians and 74.42% (96/129) of services. Of these who started using telehealth in 2020, 60.2% (236/392) of clinicians and 76.04% (73/96) of services reported that they started using telehealth in March or April. At the time of the survey, the ratio of telehealth consultations compared to face-to-face appointments varied greatly across respondents; for clinicians the majority reported telehealth consultations to be 1–25% of their total consultations (1–25% = 35.0% (163/466); 26–50% = 22.3% (104/466); 51–75% = 18.7% (87/466); 76–100% = 22.7% (106/466), “I don’t know” = 1.3% (6/466)). Similarly, the majority of managers reported that telehealth consultations were 1–25% of their services’ total consultations (1–25% = 44.4% (55/124); 26–50% = 25% (31/124); 51–75% = 11.3%. (14/124); 76–100% =15.3% (19/124); “I don’t know” = 4.0% (5/124)). Most respondents reported using telehealth consultations to monitor/review patients (97.9% of clinicians (476/486) and 96.9% services (125/128)), issue advice (94.6% of clinicians (459/485) and 95.3% services (122/128)) and for first assessments (88.1% of clinicians (428/486) and 77.3% of services (99/128)). Telehealth consultations were used to a lesser extent for triaging patients (48.1% of clinicians (232/482) and 73.6% of services (95/129)), audit (7.5% of clinicians (36/482) and 20.3% of services (26/128)) and research (8.3% of clinicians (40/482) and 11% of services (14/128)) purposes.

**Fig. 2:**
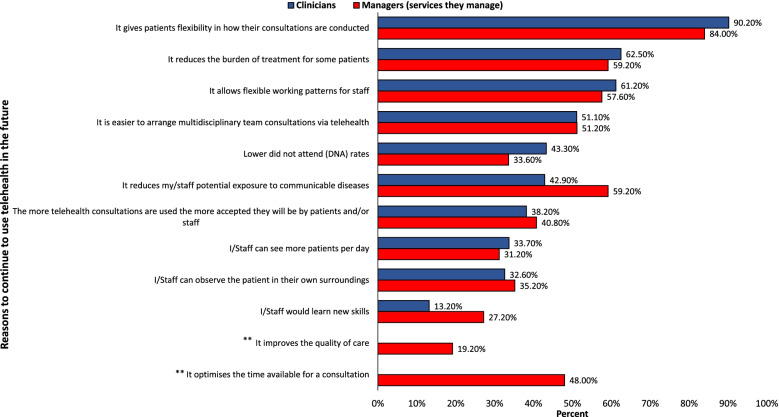
Reasons why AHP services should continue using telehealth post COVID-19 according to clinicians and managers

The majority reported that their services were planning to continue using telehealth post COVID-19 restrictions; 82.1% (400/487) of clinicians and 93.8% (120/128) of managers. The remaining clinicians reported either that their services were not planning to continue using telehealth once COVID-19 restrictions are lifted (12/487) or that they were not sure what their services’ long-term plan was (75/487). Of those clinicians whose service was not planning to continue to use telehealth, 50% (6/12) reported it was because patients were unable to use the required technology and 58.3% (7/12) reported that telehealth was not appropriate for their patients. Comparing responses across the AHP professions, most answers (4/7) stating telehealth was not appropriate for the patient group came from orthoptists, and most answers for the patients being unable to use the required technology came from physiotherapists (3/6). When asked about their opinion on their service’s long-term plan in terms of telehealth use, the majority of both clinicians (455/487, 93.4%) and managers (125/128, 97.7%) reported that they considered that their service should continue using telehealth post COVID-19. The main reasons why both clinicians and managers believed that their services should maintain telehealth after COVID-19 were that telehealth offered patients flexible consultations and that it reduced the burden of treatment for some patients (see Fig. 2).

### Barriers to telehealth

**Fig. 3:**
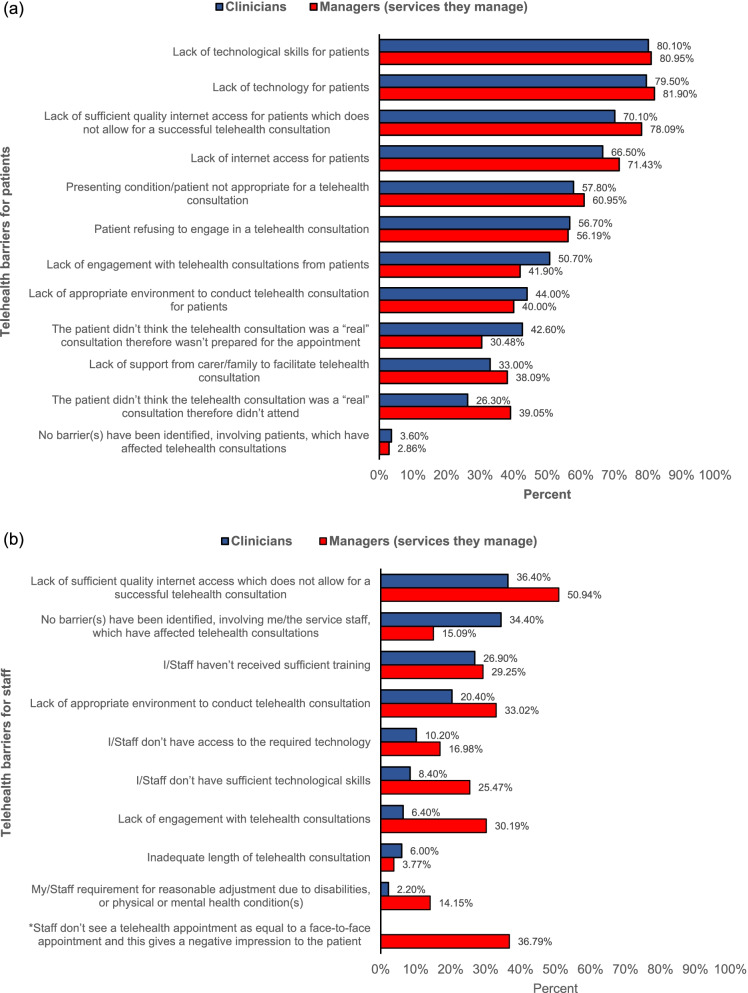
**a**) Telehealth barriers for patients; **b**) Telehealth barriers for clinicians/AHP services

For patients:

The majority of clinicians’ reported lack of technological skills for patients as the most prevalent barrier affecting the patient’s ability to conduct a telehealth consultation, alongside a lack of technology for patients, which were also the most prevalent issues reported by managers (see Fig. 3a).

For clinicians/AHP services:

The most prevalent barrier to impact the clinicians’ ability to carry out a telehealth consultation was identified as a lack of sufficient quality internet access; this barrier was also the most prevalent issue raised by managers (see Fig. 3b).

### Benefits of telehealth

**Fig. 4:**
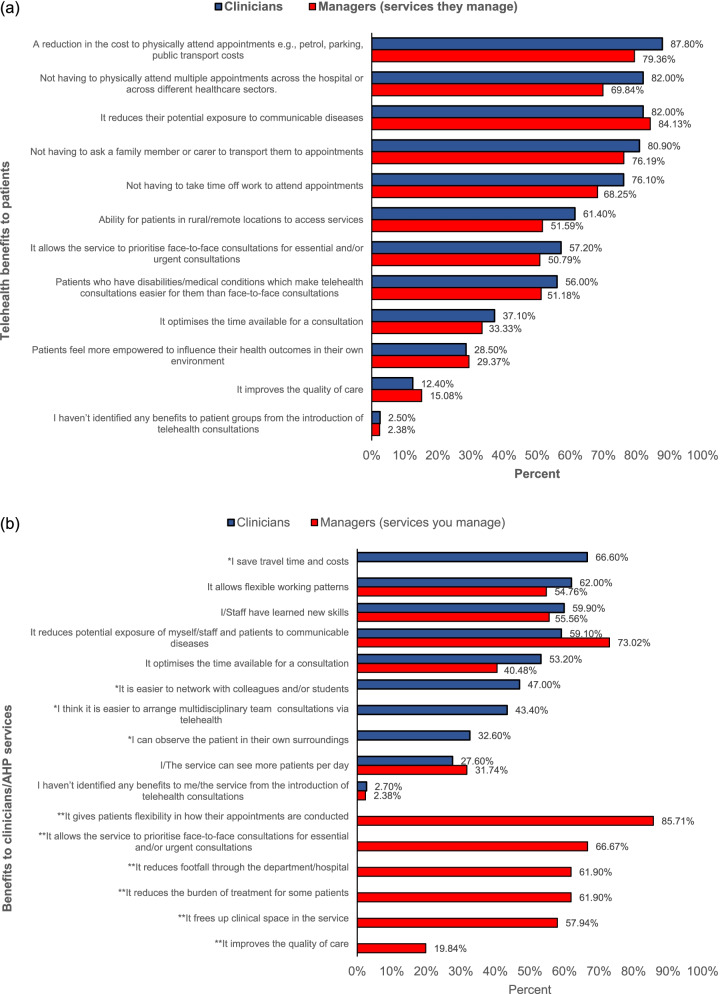
**a**) Telehealth benefits to patients; **b**) Telehealth benefits to clinicians/AHP services

For patients:

Most clinicians reported a reduction in the cost of parking and transport to attend hospital appointments as a patient benefit of telehealth consultations. With 82% of clinicians also reporting not having to physically attend appointments as a benefit to patients (see Fig. 4a).

Managers reported a reduction in potential exposure to communicable disease as the main benefit of telehealth consultation for patients, with a reduction in the cost of parking and transport as the second most prevalent response (see Fig. 4a).

For clinicians/AHP services:

Most clinicians reported that saving travel time and costs and allowing flexible working were the most important benefits of telehealth for them. Managers’ most prevalent responses when asked what the benefits of telehealth were for their service were that it gives patients flexibility in how their appointments are conducted and that it reduces potential exposure of staff to communicable diseases (Fig. 4b).

### Disadvantages of telehealth

**Fig. 5:**
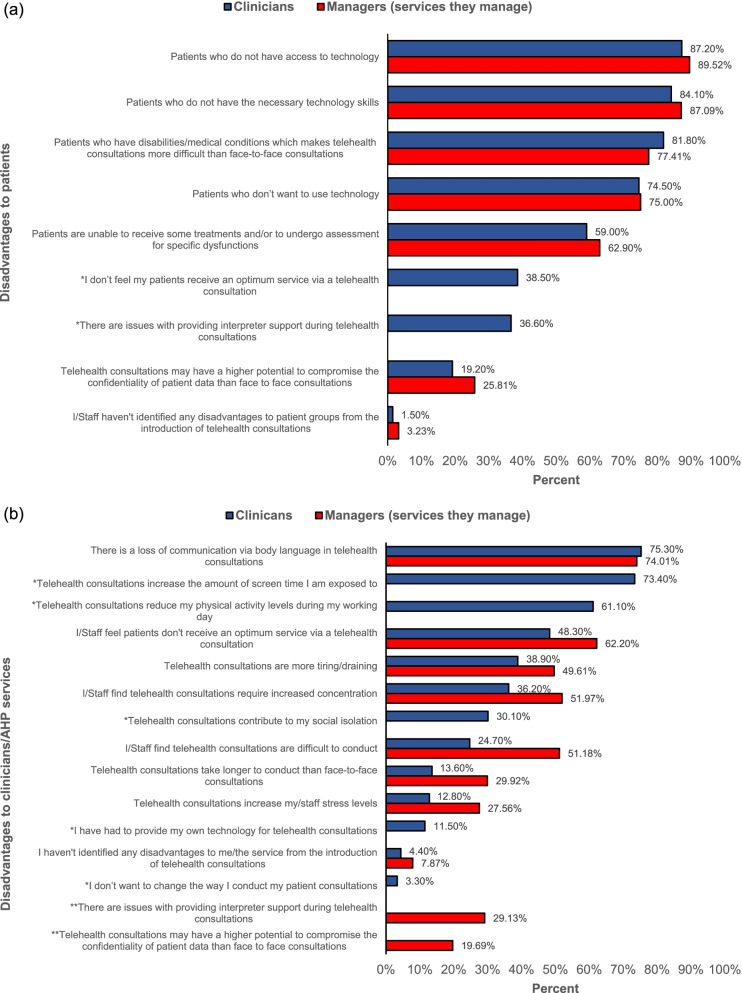
**a**) Telehealth disadvantages to patients; **b**) Telehealth disadvantages to clinicians/AHP services

For patients:

The biggest disadvantages of telehealth for patients, reported by clinicians, were not having access to technology and not having the necessary technological skills. The responses from the managers agreed with the clinicians (see Fig. 5a).

For clinicians/AHP services:

The highest reported disadvantage of telehealth for the clinician was a loss of communication via body language and an increase in exposure to screen time. A loss of communication via body language was also the most prevalent disadvantage reported by managers for their service. The second most prevalent response from managers was that staff didn’t feel the patients received an optimum service via telehealth, in contrast, clinicians reported this as the sixth most prevalent disadvantage of telehealth (see Fig. 5b).

## Discussion

The vast majority of respondents to this survey were using telehealth in their AHP services and reported that they plan to continue using telehealth post-pandemic. If the results of this study are representative of other AHP services across the NHS, it would suggest AHP services are currently in line with the NHS’ Long-Term plan of changing the “outdated” traditional model of outpatients which is unsustainable, and through service re-design and digitalisation over the next five years, avoiding up to a third of face-to-face outpatient visits [20]. Although this survey was distributed extensively to reach as many AHP staff working in the NHS as possible, the number of participants who responded to this study only represents a small number of the AHP staff employed by the NHS. Out of this, only a small proportion of the AHPs worked in Scotland and Northern Ireland. Hence our findings may not truly describe the telehealth uptake in these UK regions. Selection bias may have influenced the results of this study as current and past telehealth users may have been more inclined to participate.

While significant advancements in technology have made the widespread adoption of telehealth feasible, there are many factors to be considered when implementing a telehealth service. The pitfalls to avoid are that of neglecting people and processes and just concentrating on hardware and software [9]. Previous research reports the quality of the internet connections as a barrier for clinicians [5]. Clinicians and managers in this study also reported a lack of good quality internet access for clinicians to be the most prevalent barrier to successful telehealth consultations. This may be due to NHS services not being adequately equipped for the rapid influx of staff accessing internet services, as services introduced telehealth with very little planning, indicating that infrastructure and financial investment still needs to be considered.

Respondents identified a lack of access to technology and of technological skills for patients as barriers to conducting a telehealth consultation. There is a clear and strong relationship between groups that are digitally excluded and those at greater risk of poor health [33]. Other studies have also reported the digital literacy of patients as a barrier to successful telehealth [27, 34, 35].

Whilst it is important to ensure protection against digital exclusion when introducing telehealth consultations in AHP services, it is also vitally important to ensure the potential benefits of telehealth are recognised and utilised. Technology has the potential to reduce health inequities by enabling people to access digital health information and tools to help them to better manage their health and care. It also provides a wider platform for the NHS to reach and engage with patients from deprived areas, and it offers more convenience and choice to patients who cannot or may not want to engage with health practitioners face-to-face e.g. homeless people and people with mental health problems or debilitating illnesses [33]. It is essential that healthcare staff recognise and understand how telehealth can reduce such health inequities and may provide a more personalised approach to healthcare focusing on the delivery of services in a way that matters to the patient, aligned with their preferences and personal circumstances.

The burden of treatment can be defined as the “workload” of health care that patients must perform in response to the requirements of their healthcare providers as well as the “impact” that these practices have on patient functioning and well-being, and include attending medical appointments, undergoing investigations, taking medications, along with other aspects of self-care [36]. The reduction in time and cost has been reported to be a benefit of telehealth by patients [5]. Most clinicians and managers who participated in this study agreed telehealth would reduce the burden of treatment. Yet, when asked “*what benefits to patient groups, if any, resulted from the introduction of telehealth consultations in your service*”, more of both clinicians and managers listed at least four factors that would reduce the burden of treatment for patients. This highlights a disconnect between understanding the importance of reducing the burden of treatment and which factors might contribute to the burden of treatment. The introduction of telehealth has the potential to reduce the burden of treatment for people with long term health conditions; the impact of treatment burden for this group of patients must be understood and appreciated by healthcare staff and managers to ensure services are designed, where possible, to lessen the burden.

The highest reported disadvantage of telehealth consultations for the clinician, reported by both service managers and clinicians, was a loss of communication via body language. Appropriate training of healthcare staff to move from the traditional face-to-face mode of treatment to telehealth is essential, as the shift requires the clinician to place increased reliance on the patient to provide information that they may traditionally obtain from body language cues or via a physical examination, requiring an adaptation of existing clinical knowledge and communication skills [37]. All staff working in the NHS should be willing and receptive to digital transformation, NHS organisations should train their staff to evaluate which digital tools to select and integrate them into their clinical workflow to improve outcomes, improve user experience, and the clinicians’ working lives [9].

Also, whilst a loss of communication via body language may be considered a disadvantage, telehealth provides a level of contextual relevance that cannot be replicated within the clinic environment by allowing clinicians to observe how patients’ function within their environment. Therefore, this contextual relevance may not only have the ability to strengthen the patient-clinician relationship, but also provide advantages to clinical care that are unable to be achieved in the traditional face-to-face clinic environment [38]. Interestingly, the results seem to indicate that clinicians and managers agree that staff haven’t received sufficient training, which is in line with findings reported by AHPs in Australia, [5] but there were some disparities between the clinicians and managers. Managers reported that there was a lack of engagement among staff, and this may be due to the lack of sufficient technological skills, while clinicians reported that they engaged well with telehealth and that they already have enough skills to conduct telehealth despite the lack of training.

The second most prevalent disadvantage response reported by managers was that staff didn’t feel the patients received an optimum service via telehealth. Similar research reported that patients rated their telehealth experience highly and would like to be offered telehealth in the future [5]. This disparity may indicate a need for additional skills required of clinicians to be able to deliver care safely and effectively via the medium of telehealth [37]. It has been acknowledged that clinician acceptance is a primary determinant in the success or failure of a telehealth service [39]. If staff have a poor experience with the initial implementation of telehealth, they are less likely to accept its continued use in practice [40]. Previous studies have shown that healthcare staff report job satisfaction from having face-to-face contact with patients, reporting a change from a traditional face-to-face model of treatment delivery to telehealth as a challenge to their relationship [41]. As such, telehealth can lead to a ‘power-shift' in the empirical roles of the clinician and patient [37] which may contribute to clinician resistance and poor acceptance, but may also empower the patient. Other factors cited as key barriers to clinician acceptance towards telehealth include resistance to change, poor ICT skills amongst staff [34] and technical issues [40].

There appears to be agreement between clinicians and managers in respect to the telehealth disadvantages to patients, but they seem to disagree about disadvantages for staff. This may suggest that managers have a more negative perception of the telehealth disadvantages for staff than the staff themselves. For example, managers reported that telehealth consultations were difficult to conduct while clinicians reported they were not. This may relate to the finding that managers reported that staff don’t have sufficient skills to conduct telehealth while staff reported that their technological skills are good enough to deliver telehealth consultations.

Likewise, the responses reported for telehealth barriers for staff (Fig. 3b) are lower than those of the telehealth barriers for patients (Fig. 3a). This seems to suggest that both managers and clinicians identified more barriers to patients than to themselves/their staff. There was also agreement between clinicians and managers in the responses relating to telehealth barriers for patients, but large differences were seen for the responses for telehealth barriers for staff. This may suggest that clinicians and managers may have different perspectives on the barriers to staff.

Whilst this study did not directly measure health inequities, it was the first study to explore the barriers, advantages, disadvantages of telehealth amongst all 14 AHP professions in the UK, and the first to explore the perspectives of both AHP clinicians and AHP service managers. Further studies are required to explore the patient’s experience of telehealth consultations in AHP services.

## Conclusions

In line with previous research [27], this study indicates that if proactive measures are not taken to ensure equity, the current large-scale implementation of telehealth in NHS AHP services may increase disparities in health care access for vulnerable populations with limited digital literacy or access. Consequently, there is a danger that telehealth will be considered inappropriate, particularly if management and clinicians differ on their perceptions of barriers and disadvantages of telehealth, and thus, underutilised, negating the potential benefits of financial savings, sustainability, patient empowerment and the reduction in the burden of treatment. It is therefore imperative that there is communication and agreed understanding between clinicians and management as services re-design the way they deliver patient treatment.

### Recommendations

- AHP services must consider patient groups who may experience digital exclusion from the introduction of telehealth and ensure the design of their services and telehealth policies and guidelines allows access to face-to-face consultations, when necessary, without causing a delay in treatment, which would create an additional inequity.
- The NHS must work to ensure inclusion in digital health for vulnerable patient groups most at risk of exclusion as they move forward with the NHS Long Term Plan for digitalisation of healthcare. Investment in roles such as the “digital carer” may help negate some of these problems in the future [42].
- Healthcare staff and managers must be made aware of the potential benefits of telehealth and value its capacity to reduce the burden of treatment for patients with long term health conditions, with further education on what constitutes treatment burden.
- Effective communication and agreed understanding are required between management and clinicians to ensure the introduction of telehealth consultations is effective.

## Supplementary Information

**Additional file 1.**

## Data Availability

The datasets used and/or analysed during the current study are available from the corresponding author on reasonable request.

## Abbreviations

UK: United Kingdom’s
NHS: National Health Service
AHP: allied health professional
ICT: information and communication technology

## References

[1] Health and Care Professions Council. Registrant snapshot – 3 November 2020. [cited 2021 Nov 10]. Available from: https://www.hcpc-uk.org/about-us/insights-and-data/the-register/registrant-snapshot-1-sep-2020/

[2] Dougall D, Buck D. My role in tackling health inequalities A framework for allied health professionals. 2021. Available from: https://www.kingsfund.org.uk/publications/tackling-health-inequalities-framework-allied-health-professionals

[3] Tuckson R V, Edmunds M, Hodgkins ML. Telehealth. N Engl J Med. 2017;377(16):1585–92. 10.1056/NEJMsr1503323. 10.1056/NEJMsr1503323 29045204

[4] Hutchings R. The impact of Covid-19 on the use of digital technology in the NHS. The Nuffield Trust. 2020. Available from: https://www.nuffieldtrust.org.uk/files/2020-08/the-impact-of-covid-19-on-the-use-of-digital-technology-in-the-nhs-web-2.pdf

[5] Cottrell M, Burns CL, Jones A, Rahmann A, Young A, Sam S, et al. Sustaining allied health telehealth services beyond the rapid response to COVID-19: Learning from patient and staff experiences at a large quaternary hospital. J Telemed Telecare. 2021;27(10):615–24. Available from: 10.1177/1357633X211041517 10.1177/1357633X211041517 PMC8564219 34726993

[6] Eddison N, Healy A, Calvert S, Chockalingam N. The emergence of telehealth in orthotic services across the United Kingdom. Assist Technol. 2021:1–6. 10.1080/10400435.2021.1995531. 10.1080/10400435.2021.1995531 34663201

[7] Rowe F, Hepworth L, Howard C, Lane S (2020). Orthoptic Services in the UK and Ireland During the COVID-19 Pandemic. Br Ir Orthopt J.

[8] Sutherland R, Hodge A, Chan E, Silove N. Clinician experiences using standardised language assessments via telehealth. Int J Speech Lang Pathol. 2021;23(6):569-78. 10.1080/17549507.2021.1903079. 10.1080/17549507.2021.1903079 34000937

[9] Reddy V, Brumpton L (2021). Digital-driven service improvement during the COVID-19 pandemic. Paediatr Child Heal.

[10] Constantino JN, Sahin M, Piven J, Rodgers R, Tschida J (2020). The Impact of COVID-19 on Individuals with Intellectual and Developmental Disabilities: Clinical and Scientific Priorities. Am J Psychiatry.

[11] Lepkowsky CM, Arndt S (2019). The Internet: Barrier to health care for older adults?. Pract Innov.

[12] Peel NM, Russell TG, Gray LC (2011). Feasibility of using an in-home video conferencing system ingeriatric rehabilitation. J Rehabil Med.

[13] Dahlgren G Whitehead M. WHOLIS E89384 World Health Organization Regional Offi ce for Europe European strategies for tackling social inequities in health: Levelling up Part 2. Available from: www.euro.who.int (Accessed 2 Nov 2021).

[14] Dahlgren G, Whitehead M (2021). The Dahlgren-Whitehead model of health determinants: 30 years on and still chasing rainbows. Public Health.

[15] Michael Marmot, Peter Goldblatt JA et al. Fair Society Healthy Lives (The Marmot Review). The Institute of Health Equity. [Internet]. Available from: https://www.instituteofhealthequity.org/resources-reports/fair-society-healthy-lives-the-marmot-review

[16] Marmot M. Health equity in England: The Marmot review 10 years on. BMJ. 2020;368:1-171. 10.1136/bmj.m693 32094110

[17] Nafilyan V, Islam N, Ayoubkhani D, Gilles C, Katikireddi SV, Mathur R (2021). Ethnicity, household composition and COVID-19 mortality: a national linked data study. J R Soc Med.

[18] Joy M, Hobbs FDR, Bernal JL, Sherlock J, Amirthalingam G, Mcgagh D, et al. Excess mortality in the first COVID pandemic peak: 2020;(October):890–8.

[19] Raisi-Estabragh Z, McCracken C, Bethell MS, Cooper J, Cooper C, Caulfield MJ (2020). Greater risk of severe COVID-19 in black, asian and minority ethnic populations is not explained by cardiometabolic, socioeconomic or behavioural factors, or by 25(OH)-vitamin D status: Study of 1326 cases from the UK biobank. J Public Heal (United Kingdom).

[20] The NHS Long Term Plan. Published Online First. 2019. Available from: https://www.longtermplan.nhs.uk/

[21] Greenhalgh T, Rosen R, Shaw SE, Byng R, Faulkner S, Finlay T (2021). Planning and Evaluating Remote Consultation Services: A New Conceptual Framework Incorporating Complexity and Practical Ethics. Front Digit Heal.

[22] Leone E, Eddison N, Healy A, Royse C, Chockalingam N. Exploration of implementation, financial and technical considerations within allied health professional (AHP) telehealth consultation guidance: a scoping review including UK AHP professional bodies’ guidance. BMJ Open. 2021;11(12):e055823. Available from: http://bmjopen.bmj.com/content/11/12/e055823.abstract 10.1136/bmjopen-2021-055823 PMC8718347 34969656

[23] Share of households with internet access in the United Kingdom (UK) from 1998 to 2020 [Internet]. [cited 2020 Dec 22]. Available from: https://www.statista.com/statistics/275999/household-internet-penetration-in-great-britain/

[24] Percentage of households with mobile phones in the United Kingdom (UK) from 1996 to 2018 [Internet]. [cited 2020 Dec 22]. Available from: https://www.statista.com/statistics/289167/mobile-phone-penetration-in-the-uk/

[25] Penetration of tablet computer ownership in the United Kingdom (UK) in 2013–2020, by age [Internet]. [cited 2020 Dec 22]. Available from: https://www.statista.com/statistics/271882/tablet-owners-in-the-united-kingdom-uk-by-age/

[26] Indicator 17.8.1 - Proportion of individuals using the Internet - U.K. Indicators For The Sustainable Development Goals. [cited 2021 Nov 2]. Available from: https://sdgdata.gov.uk/17-8-1/

[27] Nouri SS, Khoong EC, Lyles CR, Karliner LS. Addressing Equity in Telemedicine for Chronic Disease Management During the Covid-19 Pandemic. NEJM Catal. 2020;1–13. Available from: https://catalyst.nejm.org/doi/full/10.1056/CAT.20.0123

[28] Lloyds Bank. UK Consumer Digital Index 2021. 2021;82. Available from: https://www.lloydsbank.com/assets/media/pdfs/banking_with_us/whats-happening/LB-Consumer-Digital-Index-2018-Report.pdf

[29] Poverty in the UK: statistics. 2021 [cited 2021 Nov 2]. Available from: https://commonslibrary.parliament.uk/research-briefings/sn07096/

[30] Lion KC, Brown JC EB (2015). Effect of telephone vs video interpretation on parent comprehension Communication, and utilization in the pediatric emergency department. JAMA Pediatr.

[31] Voils CI, Venne VL, Weidenbacher H, Sperber NDS (2018). Comparison of telephone and televideo modes for delivery of genetic counseling: a randomized trial. J Genet Couns.

[32] Davies AR, Honeyman M, Gann B. Addressing the Digital Inverse Care Law in the Time of COVID-19: Potential for Digital Technology to Exacerbate or Mitigate Health Inequalities. J Med Internet Res. 2021;23(4):e21726. 10.2196/21726. 10.2196/21726 PMC8030655 33735096

[33] NHS England. Digital Inclusion in Health and Care. [cited 2021 Nov 2]. Available from: https://www.england.nhs.uk/ltphimenu/digital-inclusion/digital-inclusion-in-health-and-care/

[34] Kruse C, Karem P, Shifflett K, Ravi Vegi L (2018). BM. Evaluating barriers to adopting telemedicine worldwide: A systematic review. J Telemed Telecare.

[35] Manganello J, Gerstner G, Pergolino K, Graham Y, Falisi ASD (2017). The relationship of health literacy with use of digital technology for health information. J Public Heal Manag Pr.

[36] Gallacher K, Jani B, Morrison D, Macdonald S, Blane D, Erwin P (2013). Qualitative systematic review of treatment burden in stroke heart failure and diabetes. BMC Med Res Methodol.

[37] Cottrell MA, Russell TG. Telehealth for musculoskeletal physiotherapy. Musculoskelet Sci Pract. 2020;48:102193. 10.1016/j.msksp.2020.102193. 10.1016/j.msksp.2020.102193 PMC7261082 32560876

[38] Cottrell MA, Hill AJ, O’Leary SP, Raymer ME, Russell TG (2018). Clinicians’ Perspectives of a Novel Home-based Multidisciplinary Telehealth Service for Patients with Chronic Spinal Pain. Int J Telerehabilitation.

[39] Wade VEliott JHillerJ, Clinician (2014). Acceptance is the key factor for sustainable telehealth services. Qual Health Res.

[40] Brewster L, Mountain G, Wessels B, Kelly C, Hawley M (2014). Factors affecting front line staff acceptance of telehealth technologies: A mixed-method systematic review. J Adv Nurs.

[41] Mair FS, Hiscock JBS (2008). Understanding factors that inhibit or promote the utilization of telecare in chronic lung disease. Chronic Illn.

[42] Open access news. The digital carer: Do the core skills for a carer need a COVID re-boot?. Open Access Goverment. [cited 2021 Nov 16]. Available from: https://www.openaccessgovernment.org/the-digital-carer-do-the-core-skills-for-a-carer-need-a-covid-re-boot/90268/

