# Supplementary material for: The potential impact of allied health professional telehealth consultations on health inequities and the burden of treatment
Source: Int J Equity Health. 2022 Jun 30;21:91. doi: 10.1186/s12939-022-01689-2 (PMC9245876; doi:10.1186/s12939-022-01689-2)
Supplement: Supplementary file 1 — Additional file 1. [file 12939_2022_1689_MOESM1_ESM.pdf]

Start of Block: Section 1 - About you

Q3.1 **Within your role do you work in a clinical capacity (i.e., directly treating patients):**

- ◯ Yes, I work solely in a clinical capacity (1)
- ◯ Yes, I work in a clinical capacity and I also work in a management capacity (2)
- ◯ No, I work solely in a management capacity (3)

Display This Question:
If Within your role do you work in a clinical capacity (i.e., directly treating patients): = Yes, I work solely in a clinical capacity
Or Within your role do you work in a clinical capacity (i.e., directly treating patients): = Yes, I work in a clinical capacity and I also work in a management capacity

Q3.2 **I work in an NHS (you do not have to be employed directly by the NHS) or local authority service as a/an:**

- ◯ Art therapist (1)
- ◯ Dietitian (2)
- ◯ Drama Therapist (3)
- ◯ Music Therapist (4)
- ◯ Occupational Therapist (5)
- ◯ Orthoptist (6)
- ◯ Orthotist (7)
- ◯ Operating Department Practitioner (8)
- ◯ Osteopath (9)
- ◯ Paramedic (10)
- ◯ Physiotherapist (11)
- ◯ Chiropodist/Podiatrist (12)
- ◯ Prosthetist (13)
- ◯ Diagnostic radiographer (14)
- ◯ Therapeutic radiographer (15)
- ◯ Speech and Language therapist (16)

Display This Question:
If Within your role do you work in a clinical capacity (i.e., directly treating patients): = No, I work solely in a management capacity

Q3.3 **What is your professional background?**

- ◯ Art therapist (1)
- ◯ Dietician (2)
- ◯ Drama Therapist (3)
- ◯ Music Therapist (4)
- ◯ Occupational Therapist (5)
- ◯ Orthoptist (6)
- ◯ Orthotist (7)
- ◯ Operating Department Practitioner (8)
- ◯ Osteopath (9)
- ◯ Paramedic (10)
- ◯ Physiotherapist (11)
- ◯ Chiropodist/Podiatrist (12)
- ◯ Prosthetist (13)
- ◯ Diagnostic radiographer (14)
- ◯ Therapeutic radiographer (15)
- ◯ Speech and Language therapist (16)
- ◯ Other (please specify) (17) ____

Display This Question:
If Within your role do you work in a clinical capacity (i.e., directly treating patients): = Yes, I work solely in a clinical capacity
Or Within your role do you work in a clinical capacity (i.e., directly treating patients): = Yes, I work in a clinical capacity and I also work in a management capacity

Q3.4 **What NHS setting(s) do you work in?** (select all that apply)

- ▢ Acute/Hospital inpatient (1)
- ▢ Acute/Hospital outpatient (2)
- ▢ Primary Care (3)
- ▢ Community service (4)
- ▢ Domiciliary (5)
- ▢ Social care (6)
- ▢ Other (please specify) (7) ____

Q3.5 **What is your current pay banding (or equivalent)?**

- Band 5 (1)
- Band 6 (2)
- Band 7 (3)
- Band 8a (4)
- Band 8b (5)
- Band 8c (6)
- Band 8d (8)
- Band 9 or above (7)
- Prefer not to say (9)

Q3.6 **How long have you worked as an AHP or managed an AHP service?**

- Less than 1 year (1)
- 1-5 years (2)
- 6-10 years (3)
- 11-15 years (4)
- 16-20 years (5)
- 21-25 years (6)
- 26-30 years (7)
- 31+ years (8)

Q3.7 **Which region of the UK do you work?** (If you work in more than one region select your primary region and answer the survey based on that region)

- ◯ England – East (1)
- ◯ England – East Midlands (2)
- ◯ England – London (3)
- ◯ England – North East (4)
- ◯ England – North West (5)
- ◯ England – South East (6)
- ◯ England – South West (7)
- ◯ England – West Midlands (8)
- ◯ England – Yorkshire and Humber (9)
- ◯ Northern Ireland (10)
- ◯ Scotland (11)
- ◯ Wales (12)

Display This Question:
If Within your role do you work in a clinical capacity (i.e., directly treating patients): = Yes, I work in a clinical capacity and I also work in a management capacity

Q3.8 As you selected that you work in both a clinical and management capacity within your role, would you like to answer questions....

- ◯ about **your** use of telehealth consultations with **your** patients (1)
- ◯ about the use of telehealth consultations with patients in the service(s) **you manage** (2)

End of Block: Section 1 - About you

Start of Block: Section 2 Telehealth use in your service (Clinician and/or Manager - services)

*

Q4.1 **Which AHP service(s) do you manage for which you have knowledge of their use of telehealth consultations with patients?** (select up to a maximum of three)

Note: For this question if you select multiple services you will be asked questions regarding availability of guidance regarding telehealth consultations, availability of training for AHPs using telehealth consultations, the impact of telehealth consultations on different patient groups and the benefits and limitations of telehealth consultations for each of the selected services. **If you prefer to only answer these questions for the service(s) which you have main responsibility for only select that/those service(s), up to a maximum of three services.** If you feel another AHP service manager would be better placed to answer the questions for a service they directly manage, please forward the survey to them.

▢ Art therapy (1)

▢ Dietetics (2)

▢ Drama Therapy (3)

▢ Music Therapy (4)

▢ Occupational Therapy (5)

▢ Orthoptics (6)

▢ Orthotics (7)

▢ Operating Department Practitioner (8)

▢ Osteopathy (9)

▢ Paramedic (10)

▢ Physiotherapy (11)

▢ Chiropody/Podiatry (12)

▢ Prosthetics (13)

▢ Radiography (14)

▢ Speech and Language therapy (15)

▢ add an additional AHP service (please specify) (16) ____

▢ add an additional AHP service (please specify) (17) ____

▢ add an additional AHP service (please specify) (18) ____

End of Block: Section 2 Telehealth use in your service (Clinician and/or Manager - services)

Start of Block: Section 2 Telehealth use in your service (Manager)

Q5.1 **Note: For this survey a telehealth consultation is defined as a telephone or video/virtual consultation with a patient. For the following questions you are asked to consider the experiences within the service you manage.**

Q5.2 **Are you currently using telehealth consultations in your ${lm://Field/1} service?**

- Yes (1)
- No (2)

Display This Question:
If Loop current: Are you currently using telehealth consultations in your ${lm://Field/1} service? = No

Q5.3 **Have you used telehealth consultations over the last 12 months in your ${lm://Field/1} service?**

- Yes (1)
- No (2)

Display This Question:
If Loop current: Have you used telehealth consultations over the last 12 months in your ${lm://Field/1} service? = No

Q5.4 **Why does your ${lm://Field/1} service not use telehealth consultations?** (select all that apply)

- We don't have the funding for the necessary equipment (1)
- We don't have the funding for the necessary software program (2)
- I don't have assurance about how staff will be protected from litigation regarding telehealth consultations (3)
- We don't have an appropriate environment to conduct telehealth consultations (4)
- The staff don't have the technological skills required (5)
- I don't think telehealth consultations are appropriate for the patients in my service (6)
- We don't have the necessary guidelines or professional standards to safely implement telehealth consultations (7)
- I think telehealth consultations would take longer to conduct than a face-to-face consultation (8)
- Staff have refused to engage in telehealth consultations (9)
- Other (please specify) (10) ____

Display This Question:
If Loop current: Have you used telehealth consultations over the last 12 months in your ${lm://Field/1} service? = No

Q5.5 **What ways would your** ${lm://Field/1} **service benefit from using/implementing telehealth consultations?** (select all that apply)

- ▢ I think it would reduce footfall through the department/hospital (1)
- ▢ I think it would give patients flexibility in how their appointments are conducted (2)
- ▢ I think it would reduce the burden of treatment for some patients (3)
- ▢ I think the service would be able to see more patients per day (4)
- ▢ I think the staff will learn new skills (5)
- ▢ I think it would allow flexible working patterns for staff (6)
- ▢ I think it would free up clinical space in the service (7)
- ▢ I think it would reduce potential exposure of staff to communicable diseases (8)
- ▢ I think it would improve the quality of care (11)
- ▢ I think it would optimise the time available for a consultation (13)
- ▢ It think it would allow the service to prioritise face-to-face consultations for essential and/or urgent consultations (15)
- ▢ Other (please specify) (9) ____
- ▢ ⊗I don't think the service would benefit from using telehealth consultations (10)

Display This Question:

If Loop current: Have you used telehealth consultations over the last 12 months in your ${lm://Field/1} service? = No

Q5.6 **What disadvantages wouldthere be to your** ${lm://Field/1} **service using/implementing telehealth consultations?** (select all that apply)

- ▢ I think telehealth consultations would take longer to conduct than face-to-face consultations (1)
- ▢ I think staff would find telehealth consultations difficult to conduct (2)
- ▢ I don't feel the patients would receive an optimum service via a telehealth consultation (3)
- ▢ I think there would be possible issues with providing interpreter support during telehealth consultations (6)
- ▢ I think it would be more difficult to ensure confidentiality of patient data in compliance with regulations and policies governing practice for telehealth consultations than for face to face consultations (7)
- ▢ Other (please specify) (4) ____
- ▢ ⊗I don't think there would be any disadvantages to my service using telehealth consultations (5)

Display This Question:
If Loop current: Have you used telehealth consultations over the last 12 months in your ${lm://Field/1} service? = No

Q5.7 **What ways would patient groups benefit(s), if any, from the introduction of telehealth consultations to your** **${lm://Field/1}** service**?** (select all that apply)

▢ Not having to physically attend multiple appointments across the hospital or across different healthcare sectors. (1)

▢ A reduction in the cost to physically attend appointments e.g., petrol, parking, public transport costs (2)

▢ Not having to take time off work to attend appointments (3)

▢ Not having to ask a family member or carer to transport them to appointments (4)

▢ It reduces their potential exposure to communicable diseases (5)

▢ I think they may feel more empowered to influence their health outcomes in their own environment (6)

▢ Patients who have disabilities/medical conditions which make telehealth consultations easier for them than face-to-face consultations (7)

▢ I think it would improve the quality of care (11)

▢ I think it would optimise the time available for a consultation (12)

▢ Ability for patients in rural/remote locations to access services (10)

▢ It think it would allow the service to prioritise face-to-face consultations for essential and/or urgent consultations (13)

▢ Other (please specify) (8) ____

▢ ⊗I don't think there would be any benefits to patient groups from the introduction of telehealth consultations (9)

Display This Question:
If Loop current: Have you used telehealth consultations over the last 12 months in your ${lm://Field/1} service? = No

Q5.8 **What disadvantage(s) would there be to** patient groups**, if any, from the introduction of telehealth consultations to your ${lm://Field/1} service?** (select all that apply)

- ▢ Patients who have disabilities/medical conditions which make telehealth consultations more difficult than face-to-face consultations e.g., visual and hearing impairments, patients with learning disabilities (1)
- ▢ Patients who don't have access to technology (2)
- ▢ Patients who don't have the necessary technology skills (3)
- ▢ Patient not wanting to use technology (4)
- ▢ I don't think patients would receive an optimum service via a telehealth consultation (8)
- ▢ I think there might be issues with providing interpreter support during telehealth consultations (9)
- ▢ I think patients might not be unable to receive some treatments and/or to undergo assessment for specific dysfunctions (10)
- ▢ I think it would be more difficult to ensure confidentiality of patient data in compliance with regulations and policies governing practice for telehealth consultations than for face to face consultations (11)
- ▢ Other (please specify) (6) ____
- ▢ ⊗I haven't identified any disadvantages to patient groups from the introduction of telehealth consultations (7)

Display This Question:

If Loop current: Are you currently using telehealth consultations in your ${lm://Field/1} service? = No

And Loop current: Have you used telehealth consultations over the last 12 months in your ${lm://Field/1} service? = Yes

Q5.9 **Why has your** ${lm://Field/1} **service stopped using telehealth consultations?** (select all that apply)

- ☐ Telehealth consultations are not appropriate for our patient groups (1)
- ☐ Telehealth consultations have resulted in poorer clinical outcomes for patients (2)
- ☐ The patients didn't like it (3)
- ☐ The patients couldn't use the required technology to facilitate the conversation (4)
- ☐ Telehealth consultations have directly resulted in patient complaints (5)
- ☐ Telehealth consultations negatively affected key performance indicators (KPIs) (6)
- ☐ I don't have assurance about how staff will be protected from litigation regarding telehealth consultations (7)
- ☐ Telehealth consultations took longer than face-to-face consultations (8)
- ☐ The patients couldn't use the required technology to facilitate the conversation (9)
- ☐ The technology wasn't reliable (10)
- ☐ The software wasn't reliable (11)
- ☐ The staff tried it and didn't like it (12)
- ☐ The staff didn't have the skills necessary to conduct a telehealth appointment properly (13)
- ☐ The staff do not have the attributes required to adapt to a new way of conducting consultations (e.g., adaptability, preparedness, willingness to try new things, acceptance that things might not go to plan) (14)
- ☐ Telehealth consultations increased stress levels amongst the staff (15)
- ☐ We don't get the same funding for a telehealth consultation compared to a face-to-face consultation (16)
- ☐ The referrers stipulated that face-to-face consultations are preferred. (17)
- ☐ Other (please specify) (18) ____

Display This Question:
If Loop current: Are you currently using telehealth consultations in your ${lm://Field/1} service? = Yes
Or Loop current: Have you used telehealth consultations over the last 12 months in your ${lm://Field/1} service? = Yes

Q5.10 **What setting(s) has your** ${lm://Field/1} **service used telehealth consultations over the last 12 months?** (select all that apply)

- ▢ Acute/Hospital outpatient (1)
- ▢ Primary Care (2)
- ▢ Community service (3)
- ▢ Domiciliary (4)
- ▢ Social care (5)
- ▢ Other (please specify) (6) ____

Display This Question:
If Loop current: Are you currently using telehealth consultations in your ${lm://Field/1} service? = Yes
Or Loop current: Have you used telehealth consultations over the last 12 months in your ${lm://Field/1} service? = Yes

Q5.11 **When did your** ${lm://Field/1} **service start using telehealth consultations?**

	Month	Year
Please Select: (1)	▼ January (1 ... December (12)	▼ 1980 (1 ... 2021 (42)

Display This Question:
If Loop current: Are you currently using telehealth consultations in your ${lm://Field/1} service? = Yes
Or Loop current: Have you used telehealth consultations over the last 12 months in your ${lm://Field/1} service? = Yes

Q5.12 **For what types of consultations does/has your** ${lm://Field/1} **service use/used telehealth consultations?**

	Yes, always by telephone (1)	Yes, always by video (2)	Yes, by either telephone or video (3)	No (4)	Not applicable (5)
To triage patients (1)	○	○	○	○	○
First assessment (2)	○	○	○	○	○
Monitor/Follow up/Review (3)	○	○	○	○	○
Issuing of advice (4)	○	○	○	○	○
Issuing a piece of equipment or a medical device (5)	○	○	○	○	○
Research purposes (6)	○	○	○	○	○
Audit purposes (7)	○	○	○	○	○

Display This Question:
If Loop current: Are you currently using telehealth consultations in your ${lm://Field/1} service? = Yes

Q5.13 **Currently, what ratio is your** ${lm://Field/1} **service's telehealth consultations compared to face-to-face consultations?**

- ◯ 1-25% of my consultations are telehealth consultations (1)
- ◯ 26-50% of my consultations are telehealth consultations (2)
- ◯ 51-75% of my consultations are telehealth consultations (3)
- ◯ 76-100% of my consultations are telehealth consultations (4)
- ◯ I don't know (5)

Display This Question:
If Loop current: Are you currently using telehealth consultations in your ${lm://Field/1} service? = Yes
Or Loop current: Have you used telehealth consultations over the last 12 months in your ${lm://Field/1} service? = Yes

Q5.14 **Does your** ${lm://Field/1} **service plan to continue using telehealth (in some capacity) post COVID-19, when there are no restrictions on face-to-face consultations?**

- ◯ Yes (1)
- ◯ No (2)
- ◯ I don't know (3)

Display This Question:
If Loop current: Does your ${lm://Field/1} service plan to continue using telehealth (in some capacity) post COVID... = No

Q5.15 **Why does your** ${lm://Field/1} **service not intend to continue using telehealth consultations post COVID-19?** (select all that apply)

- ☐ Telehealth consultations are not appropriate for our patient groups (1)
- ☐ Telehealth consultations have resulted in poorer clinical outcomes for patients (2)
- ☐ The patients didn't like it (3)
- ☐ The patients couldn't use the required technology to facilitate the conversation (4)
- ☐ Telehealth consultations have directly resulted in patient complaints (5)
- ☐ Telehealth consultations negatively affected key performance indicators (KPIs) (6)
- ☐ Telehealth consultations took longer than face-to-face consultations (7)
- ☐ The patients couldn't use the required technology to facilitate the conversation (8)
- ☐ The technology wasn't reliable (9)
- ☐ The software wasn't reliable (10)
- ☐ The staff tried it and didn't like it (11)
- ☐ The staff don't have the skills necessary to conduct a telehealth appointment properly (12)
- ☐ The staff do not have the attributes required to adapt to a new way of conducting consultations (e.g., adaptability, preparedness, willingness to try new things, acceptance that things might not go to plan) (13)
- ☐ Telehealth consultations increased stress levels amongst the staff (14)
- ☐ We don't get the same funding for a telehealth consultation compared to a face-to-face consultation (15)
- ☐ The referrers stipulated that face-to-face consultations are preferred (16)
- ☐ Other (please specify) (17) ____

Display This Question:

If Loop current: Are you currently using telehealth consultations in your ${lm://Field/1} service? = Yes

Or Loop current: Have you used telehealth consultations over the last 12 months in your ${lm://Field/1} service? = Yes

Q5.16 **Do you think your** ${lm://Field/1} **service should continue using telehealth (in some capacity) post COVID-19, when there are no restrictions on face-to-face consultations?**

- ○ Yes (1)
- ○ No (2)
- ○ I don't know (3)

Display This Question:
If Loop current: Do you think your ${lm://Field/1} service should continue using telehealth (in some capacity) pos... = Yes

Q5.17 **Why do you think your** ${lm://Field/1} **service should continue using telehealth (in some capacity) post COVID-19?** (select all that apply)

- ☐ Staff would learn new skills (1)
- ☐ It is easier to arrange multidisciplinary team (MDT) consultations via telehealth (2)
- ☐ Staff can observe the patient in their own surroundings (3)
- ☐ Staff can see more patients per day (4)
- ☐ It allows flexible working patterns for staff (5)
- ☐ It reduces potential exposure to communicable diseases for staff (6)
- ☐ It gives patients flexibility in how their consultations are conducted (7)
- ☐ It reduces the burden of treatment for some patients (8)
- ☐ The more telehealth consultations are used the more accepted they will be by patients and/or staff (9)
- ☐ It improves the quality of care (11)
- ☐ It optimises the time available for a consultation (12)
- ☐ Lower did not attend (DNA) rates (13)
- ☐ Other (please specify) (10) ____

Display This Question:
If Loop current: Do you think your ${lm://Field/1} service should continue using telehealth (in some capacity) pos... = No

Q5.18 **Why don't you think your** ${lm://Field/1} **service should continue using telehealth (in some capacity) post COVID-19?** (select all that apply)

- ☐ Telehealth consultations take longer to conduct than face-to-face consultations (1)
- ☐ Staff find telehealth consultations difficult to conduct (2)
- ☐ Telehealth consultations increase staff stress levels (3)
- ☐ Staff don't feel the patients receive an optimum service via a telehealth consultation (4)
- ☐ Other (please specify) (5) ____

Display This Question:
If Loop current: Are you currently using telehealth consultations in your ${lm://Field/1} service? = Yes
Or Loop current: Have you used telehealth consultations over the last 12 months in your ${lm://Field/1} service? = Yes

Q5.21 **What benefit(s) to your** ${lm://Field/1} **service, if any, have resulted from the introduction of telehealth consultations?** (select all that apply)

- ☐ It reduces footfall through the department/hospital (1)
- ☐ It gives patients flexibility in how their appointments are conducted (2)
- ☐ It reduces the burden of treatment for some patients (3)
- ☐ The service can see more patients per day (4)
- ☐ The staff have learned new skills   (5)
- ☐ It allows flexible working patterns for staff (6)
- ☐ It frees up clinical space in the service (7)
- ☐ It reduces potential exposure of staff and patients to communicable diseases (8)
- ☐ It improves the quality of care (11)
- ☐ It optimises the time available for a consultation (12)
- ☐ It allows the service to prioritise face-to-face consultations for essential and/or urgent consultations (13)
- ☐ Other (please specify) (9) ____
- ☐ ⊗I haven't identified any benefits to the service from the introduction of telehealth consultations (10)

Display This Question:
If Loop current: Are you currently using telehealth consultations in your ${lm://Field/1} service? = Yes
Or Loop current: Have you used telehealth consultations over the last 12 months in your ${lm://Field/1} service? = Yes

Q5.19 **What benefit(s) to patient groups, if any, have resulted from the introduction of telehealth consultations in your** ${lm://Field/1} **service?** (select all that apply) (i.e., has the introduction of telehealth consultations reduced obstacles that interfere with a patient's health care or reduced the burden of healthcare experienced by patients?)

- ▢ Not having to physically attend multiple appointments across the hospital or across different healthcare sectors. (1)
- ▢ A reduction in the cost to physically attend appointments e.g., petrol, parking, public transport costs (2)
- ▢ Not having to take time off work to attend appointments (3)
- ▢ Not having to ask a family member or carer to transport them to appointments (4)
- ▢ It reduces their potential exposure to communicable diseases (5)
- ▢ They feel more empowered to influence their health outcomes in their own environment (6)
- ▢ Patients who have disabilities/medical conditions which make telehealth consultations easier for them than face-to-face consultations (7)
- ▢ It improves the quality of care (10)
- ▢ It optimises the time available for a consultation (12)
- ▢ Ability for patients in rural/remote locations to access services (13)
- ▢ It allows the service to prioritise face-to-face consultations for essential and/or urgent consultations (14)
- ▢ Other (please specify) (8) ____
- ▢ ⊗I haven’t identified any benefits to patient groups from the introduction of telehealth consultations (9)

Display This Question:

If Loop current: Are you currently using telehealth consultations in your ${lm://Field/1} service? = Yes

Or Loop current: Have you used telehealth consultations over the last 12 months in your ${lm://Field/1} service? = Yes

Q5.20 **What disadvantage(s) to patient groups, if any, have resulted from the introduction of telehealth consultations in your** ${lm://Field/1} **service?** (select all that apply)

▢ Patients who have disabilities/medical conditions which makes telehealth consultations more difficult than face-to-face consultations e.g., visual and hearing impairments, patients with learning disabilities (1)

▢ Patients who do not have access to technology (2)

▢ Patients who do not have the necessary technology skills (3)

▢ Patients who don't want to use technology (4)

▢ Staff found patients were unable to receive some treatments and/or to undergo assessment for specific dysfunctions (8)

▢ Telehealth consultations may have a higher potential to compromise the confidentiality of patient data in compliance with regulations and policies governing practice than face to face consultations (9)

▢ Other (please specify) (6) ____

▢ ⊗I haven't identified any disadvantages to patient groups from the introduction of telehealth consultations (7)

Display This Question:
If Loop current: Are you currently using telehealth consultations in your ${lm://Field/1} service? = Yes
Or Loop current: Have you used telehealth consultations over the last 12 months in your ${lm://Field/1} service? = Yes

Q5.22 **What disadvantage(s) to your** ${lm://Field/1} **service, if any, have resulted from the introduction of telehealth consultations in your service?** (select all that apply)

▢ Telehealth consultations take longer to conduct than face-to-face consultations (1)

▢ Staff find telehealth consultations difficult to conduct (2)

▢ Telehealth consultations increase staff stress levels (3)

▢ Staff find telehealth consultations more tiring/draining (7)

▢ Staff find there is a loss of communication via body language in telehealth consultations (8)

▢ Staff find telehealth consultations require increased concentration (9)

▢ Staff feel the patients don't receive an optimum service via a telehealth consultation (4)

▢ There are issues with providing interpreter support during telehealth consultations (10)

▢ Telehealth consultations may have a higher potential to compromise the confidentiality of patient data in compliance with regulations and policies governing practice than face to face consultations (11)

▢ Other (please specify) (5) ____

▢ ⊗I haven't identified any disadvantages to the service from the introduction of telehealth consultations (6)

End of Block: Section 2 Telehealth use in your service (Manager)

Start of Block: Section 3 Telehealth consultation guidance in your service (manager)

Q6.1 **Has your ${lm://Field/1} service implemented guidance on how telehealth consultations should be set-up?**

○ Yes (1)

○ No (2)

○ I don't know (3)

Display This Question:
If Loop current: Has your ${lm://Field/1} service implemented guidance on how telehealth consultations should be s... = No

Q6.2 **Why haven't you implemented guidance to inform telehealth consultations in your ${lm://Field/1} service?** (select all that apply)

▢ I don't think my service needs guidance for telehealth consultations (1)

▢ I don't have access to guidance to inform telehealth consultations in my service (2)

▢ I am in the process of writing guidance for telehealth consultations in my service (3)

▢ I didn't understand the available guidance (4)

▢ The available guidance is unclear/ambiguous, where it is left to me/my staff to decide (5)

▢ The guidance lacks information or details on certain areas (for example, guidance on which patient groups are suitable for telehealth consultations, how to ensure minimal treatment delays for patients who have had an unsuccessful telehealth consultation, patient confidentiality and information governance or guidance on the use of technology to facilitate telehealth consultations) (6)

▢ Other (please specify) (7) ____

Skip To: End of Block If Condition: Selected Count Is Greater Than or Equal to 1. Skip To: End of Block.

Display This Question:
If Loop current: Has your ${lm://Field/1} service implemented guidance on how telehealth consultations should be s... = Yes

Q6.3 **Who produced the ${lm://Field/1} service guidance?** (select all that apply)

- [ ] The ${lm://Field/1} professional body (5)
- [ ] Another AHP professional body (1)
- [ ] Another professional body (not AHP) (6)
- [ ] Me, as the ${lm://Field/1} service manager (3)
- [ ] My department/team (7)
- [ ] My NHS Trust (2)
- [ ] Other (please specify) (4) ____

Q6.4 **Do you think that your ${lm://Field/1} service guidance on telehealth consultations:**

	Yes (1)	No (2)	I don't know (3)
has areas of ambiguity, where it is left to you to decide (1)	o	o	o
lacks information or details on certain areas (for example, guidance on which patient groups are suitable for telehealth consultations, how to ensure minimal treatment delays for patients who have had an unsuccessful telehealth consultation, patient confidentiality and information governance or guidance on the use of technology to facilitate telehealth consultations) (2)	o	o	o

Display This Question:
If Loop current: Do you think that your ${lm://Field/1} service guidance on telehealth consultations: [Yes] (Count) >= 1

Q6.5 **What area(s) of the ${lm://Field/1} service guidance are ambiguous or lack information?** (select all that apply)

- [ ] Recommended consultation length (1)
- [ ] How to conduct an assessment without physical contact (2)
- [ ] Which patients are suitable for a telehealth consultation (3)
- [ ] Which treatment/interventions are appropriate for a telehealth consultation (4)
- [ ] How the clinician is protected from litigation should a telehealth consultation go wrong (5)
- [ ] How to deal with an emergency during a telehealth consultation (6)
- [ ] Training requirements for telehealth consultations (7)
- [ ] Other (please specify) (8) ____

Q6.6 **Does the ${lm://Field/1} service guidance advise on:**

	Yes (1)	No (2)	I don't know (3)	Not applicable (4)
How to triage a referral for a telehealth consultation? (1)	○	○	○	○
The appropriate environment for staff to conduct a telehealth consultation? (2)	○	○	○	○
The appropriate environment for a patient to conduct a telehealth consultation? (3)	○	○	○	○
The appropriate technology for staff to conduct a telehealth consultation? (4)	○	○	○	○
The appropriate technology for a patient to conduct a telehealth consultation? (5)	○	○	○	○
The medical conditions which can be treated via a telehealth consultation? (6)	○	○	○	○
The type of consultation which can be conducted via a telehealth consultation (e.g. first assessment, follow up/review, issue of advice, issue of a piece of equipment or a medical device etc.)? (7)	○	○	○	○
The recommended consultation length required for each consultation type? (8)	○	○	○	○
Which patient groups might not be able to engage via a telehealth consultation? (9)	○	○	○	○
How to ensure patient groups who might not be able to engage via a telehealth consultation are identified and appointed appropriately ensuring they do not have to wait longer that another patient with the same clinical urgency? (10)	○	○	○	○
How to conduct a telehealth consultation with a patient who requires an interpreter? (11)	○	○	○	○
How to conduct a telehealth consultation with a patient who has a physical, mental, learning or communication disability? (12)	○	○	○	○
How to conduct a telehealth consultation with patients from different age groups? (13)	○	○	○	○

Q6.7 **Does the ${lm://Field/1} service guidance advise on how patients with physical, mental, learning or communication disabilities or those who require an interpreter should be identified, triaged and supported to ensure they are given an appropriate consultation to meet their needs?**

○ Yes, these patient groups are not offered a telehealth consultation only a face-to-face consultation. (1)

○ Yes, we offer these patients a telehealth consultation first and if this is unsuccessful, they are then offered face-to face consultation. (2)

○ No, the guidance does not offer advice on which consultation method these patients should receive. (3)

○ I don't know (5)

○ Other (please specify) (4) ____

End of Block: Section 3 Telehealth consultation guidance in your service (manager)

Start of Block: Section 4 Telehealth consultation training in your service (manager)

Q7.1 **Have the Allied Health Professionals (AHPs) in your ${lm://Field/1} service been given training on the following?**

	Yes (1)	No, but I don't think staff need training (2)	No, and I think staff need training (3)
How to use the hardware required for conducting telehealth consultations? (1)	○	○	○
How to use the software required for conducting telehealth consultations? (2)	○	○	○
How to conduct a risk assessment prior to starting a telehealth consultation? (for example, assessing the patient's environment before commencing a telehealth consultation) (3)	○	○	○
How to conduct a clinical assessment via a telehealth consultation? (4)	○	○	○
How to provide a treatment intervention via a telehealth consultation? (5)	○	○	○
How to ensure patient confidentially when conducting telehealth consultations? (6)	○	○	○
How to get feedback from patients and their families about telehealth consultations? (7)	○	○	○
How to deal with an emergency during a telehealth consultation (8)	○	○	○

End of Block: Section 4 Telehealth consultation training in your service (manager)

Start of Block: Section 5 Factors affecting telehealth consultations in your service (manager)

Q8.1 **What barriers involving your ${lm://Field/1} service's staff, if any, have affected telehealth consultations? (select all that apply)** (select all that apply)

- ☐ Staff haven't received sufficient training (1)
- ☐ Staff don't have access to the required technology (2)
- ☐ Staff don't have sufficient technological skills (3)
- ☐ Lack of sufficient quality internet access (i.e., Staff have internet access, but the quality of the connection does not allow for a successful telehealth consultation) (4)
- ☐ Staff don't have an appropriate environment to conduct telehealth consultation (5)
- ☐ Staff lack of engagement with telehealth consultations (6)
- ☐ The staff don't see a telehealth appointment as equal to a face-to-face appointment and this gives a negative impression to the patient. (7)
- ☐ Inadequate length of telehealth consultation (8)
- ☐ Staff requirement for reasonable adjustment (adjustments required due to disabilities, or physical or mental health condition(s)) (9)
- ☐ Other (please specify) (10) ____
- ☐ ⊗No barrier(s) have been identified, involving the service's staff, which have affected telehealth consultations (11)

Q8.2 **What barriers involving your ${lm://Field/1} service's patients, if any, have affected telehealth consultations?** (select all that apply)

- ▢ Patient refusing to engage in a telehealth consultation (1)
- ▢ Lack of technology for patients (2)
- ▢ Lack of technological skills for patients (3)
- ▢ Lack of internet access for patients (4)
- ▢ Lack of sufficient quality internet access for patients (patients may have internet access but the quality of the connection does not allow for a successful telehealth consultation) (5)
- ▢ Lack of appropriate environment to conduct telehealth consultation for patients (6)
- ▢ Lack of engagement with telehealth consultations from patients (7)
- ▢ Lack of support from carer/family to facilitate telehealth consultation (13)
- ▢ Presenting condition/patient not appropriate for a telehealth consultation (8)
- ▢ The patient didn't think the telehealth consultation was a "real" consultation therefore wasn't prepared for the appointment (9)
- ▢ The patient didn't think the telehealth consultation was a "real" consultation therefore didn't attend (10)
- ▢ Other (please specify) (11) ____
- ▢ ⊗No barrier(s) have been identified, involving the service's patients, which have affected telehealth consultations (12)

End of Block: Section 5 Factors affecting telehealth consultations in your service (manager)

Start of Block: Section 6 Use of Make Every Contact Count (MECC) in your service (manager)

Q9.1 **Within your ${lm://Field/1} service do the AHPs routinely have healthy behavior conversations in line with Make Every Contact Count (MECC) with your patients? e.g., conversations on weight loss, smoking and exercise.**

- ◯ Yes (1)
- ◯ No (2)
- ◯ I don't know (3)

Display This Question:

If Loop current: Within your ${lm://Field/1} service do the AHPs routinely have healthy behavior conversations in... = Yes

Q9.2 **Does the use of telehealth consultations in your ${lm://Field/1} service affect MECC conversations?**

○ Yes, it makes them easier (1)

○ Yes, it makes them harder (2)

○ No (3)

○ I don't know (4)

End of Block: Section 6 Use of Make Every Contact Count (MECC) in your service (manager)

Start of Block: Section 7 Consultation payment system in your service

Q10.1 **Do you have budgetary responsibility for your ${lm://Field/1} service?**

○ Yes (1)

○ No (2)

Display This Question:

If Loop current: Do you have budgetary responsibility for your ${lm://Field/1} service? = Yes

Q10.2 **Does your ${lm://Field/1} service get paid (i.e., the way they are funded via the local Clinical Commissioning Group (CCG)) or Health Board (HB) or Health and Social Care Board (HSCB) a different rate for a telehealth consultation compared to a face-to-face consultation for the same purpose?**

○ No, the service gets paid the same rate (1)

○ Yes, the service gets paid more for a face-to-face consultation compared to a telehealth consultation (2)

○ Yes, the service gets paid more for a telehealth consultation compared to a face-to-face consultation (3)

○ There have not been any discussions/agreements with the CCG regarding the rate for telehealth consultations, so payment have remained the same (4)

○ I do not know (5)

○ Other (please specify) (6) ____

End of Block: Section 7 Consultation payment system in your service

---

Start of Block: Section 2 Telehealth use in your service (Clinician - Part 1)

Q11.1 **Note: For this survey a telehealth consultation is defined as a telephone or video/virtual consultation with a patient.. For the following questions you are asked to give your personal experience within your clinical role.**

Q11.2 **Are you currently using telehealth consultations?**

○ Yes (1)

○ No (2)

Display This Question:

If Are you currently using telehealth consultations? = No

Q11.3 **Have you used telehealth consultations over the last 12 months?**

○ Yes (1)

○ No (2)

End of Block: Section 2 Telehealth use in your service (Clinician - Part 1)

Start of Block: Section 2 Telehealth use in your service (Clinician - has not used telehealth)

Q12.1 **Why do you not use telehealth consultations?** (select all that apply)

▢ ⊗My service hasn't implemented telehealth consultations (1)

▢ I don't have assurance about how I will be protected from litigation regarding telehealth consultations (2)

▢ I don't have an appropriate environment to conduct telehealth consultation (3)

▢ I don't have the necessary equipment (4)

▢ I don't have the necessary software program (5)

▢ I don't have the technological skills required (6)

▢ I haven't received the necessary training (7)

▢ I don't think telehealth consultations are appropriate for my patients (8)

▢ I think telehealth consultations would take longer to conduct than a face-to-face consultation (9)

▢ I have a disability/medical condition that prevents me from using telehealth consultations (10)

▢ Other (please specify) (11) ____

Q12.2 **What disadvantages would there be to you using/implementing telehealth consultations?** (select all that apply)

- ▢ I think telehealth consultations would take longer to conduct than face-to-face consultations (1)
- ▢ I think I would find telehealth consultations difficult to conduct (2)
- ▢ I think telehealth consultations would increase my stress levels (3)
- ▢ I think telehealth consultations would be more tiring/draining (12)
- ▢ I think there would be a loss of communication via body language in telehealth consultations (13)
- ▢ I think telehealth consultations would require increased concentration (14)
- ▢ I think telehealth consultations would contribute to social isolation (11)
- ▢ I don’t feel I could give my patients an optimum service via a telehealth consultation (4)
- ▢ I don’t want to change the way I conduct my patient consultations (5)
- ▢ I think telehealth consultations would reduce my physical activity levels during my working day (6)
- ▢ I think telehealth consultations would increase the amount of screen time I am exposed too (7)
- ▢ I would have to provide my own technology for telehealth consultations (8)
- ▢ Other (please specify) (9) ____
- ▢ ⊗I don’t think there would be any disadvantages to me using telehealth consultations (10)

Q12.3 **What ways would you benefit from using telehealth consultations?** (select all that apply)

- ☐ I would learn new skills (1)
- ☐ I think it would be easier to arrange multidisciplinary team (MDT) consultations via telehealth (2)
- ☐ I could observe the patient in their own surroundings (3)
- ☐ I would be able to see more patients per day (4)
- ☐ It would allow flexible working patterns (5)
- ☐ It would reduce my potential exposure to communicable diseases (6)
- ☐ I would save travel time and costs (9)
- ☐ I think it would be easier to network with colleagues and/or students (11)
- ☐ It optimises the time available for a consultation (13)
- ☐ Other (please specify) (7) ____
- ☐ ⊗I don’t think I would benefit from using telehealth consultations (8)

Q12.4 **What ways would patient groups benefit(s), if any, from the introduction of telehealth consultations?** (select all that apply)

- ▢ Not having to physically attend multiple appointments across the hospital or across different healthcare sectors. (1)
- ▢ A reduction in the cost to physically attend appointments e.g., petrol, parking, public transport costs (2)
- ▢ Not having to take time off work to attend appointments (3)
- ▢ Not having to ask a family member or carer to transport them to appointments (4)
- ▢ It reduces their potential exposure to communicable diseases (5)
- ▢ I think they may feel more empowered to influence their health outcomes in their own environment (6)
- ▢ Patients who have disabilities/medical conditions which make telehealth consultations easier for them than face-to-face consultations (7)
- ▢ I think it would improve the quality of care (11)
- ▢ I think it would optimise the time available for a consultation (12)
- ▢ Ability for patients in rural/remote locations to access services (10)
- ▢ I think it would allow the service to prioritise face-to-face consultations for essential and/or urgent consultations (13)
- ▢ Other (please specify) (8) ____
- ▢ ⊗I don't think there would be any benefits to patient groups from the introduction of telehealth consultations (9)

Q12.5 **What disadvantage(s) would there be to** patient groups**, if any, from the introduction of telehealth consultations?** (select all that apply)

- ▢ Patients who have disabilities/medical conditions which make telehealth consultations more difficult than face-to-face consultations e.g., visual and hearing impairments, patients with learning disabilities (1)
- ▢ Patients who don't have access to technology (2)
- ▢ Patients who don't have the necessary technology skills (3)
- ▢ Patient not wanting to use technology (4)
- ▢ I don’t think my patients would receive an optimum service via a telehealth consultation (8)
- ▢ I think there might be issues with providing interpreter support during telehealth consultations (9)
- ▢ I think patients might not be unable to receive some treatments and/or to undergo assessment for specific dysfunctions (10)
- ▢ I think it would be more difficult to ensure confidentiality of patient data in compliance with regulations and policies governing practice for telehealth consultations than for face to face consultations (11)
- ▢ Other (please specify) (6) ____
- ▢ ⊗I haven’t identified any disadvantages to patient groups from the introduction of telehealth consultations (7)

End of Block: Section 2 Telehealth use in your service (Clinician - has not used telehealth)

Start of Block: Section 2 Telehealth use in your service (Clinician - has used telehealth)

Display This Question:

If Are you currently using telehealth consultations? = No

Q13.1 **Why have you stopped using telehealth consultations?** (select all that apply)

- [ ] My service has stopped using telehealth consultations (1)
- [ ] I don't have assurance about how I will be protected from litigation regarding telehealth consultations (2)
- [ ] The patients didn't like it (3)
- [ ] I didn't like it (4)
- [ ] The patients couldn't use the required technology to facilitate the consultation (5)
- [ ] Telehealth consultations have resulted in poorer clinical outcomes for patients (6)
- [ ] Telehealth consultations took longer than face-to-face consultations (7)
- [ ] I didn't understand how to conduct a telehealth appointment properly (8)
- [ ] The technology wasn't reliable (9)
- [ ] The software wasn't reliable (10)
- [ ] Other (please specify) (11) ____

Q13.2 **What setting(s) have you used telehealth consultations over the last 12 months?** (select all that apply)

- [ ] Acute/Hospital outpatient (1)
- [ ] Primary Care (2)
- [ ] Community service (3)
- [ ] Domiciliary (4)
- [ ] Social care (5)
- [ ] Other (please specify) (6) ____

Q13.3 **When did you start using telehealth consultations?**

	Month	Year
Please Select: (1)	▼ January (1 ... December (12)	▼ 1980 (1 ... 2021 (42)

Q13.4 **For what types of consultations do/have you use/used telehealth consultations?**

	Yes (1)	No (2)	Not applicable (3)
To triage patients (1)	○	○	○
First assessment (2)	○	○	○
Monitor/Follow up/Review (3)	○	○	○
Issuing of advice (4)	○	○	○
Issuing a piece of equipment or a medical device (5)	○	○	○
Research purposes (6)	○	○	○
Audit purposes (7)	○	○	○

Display This Question:
If Are you currently using telehealth consultations? = Yes

Q13.5 **Currently, what ratio are your telehealth consultations compared to face-to-face consultations?**

○ 1-25% of my consultations are telehealth consultations (1)

○ 26-50% of my consultations are telehealth consultations (2)

○ 51-75% of my consultations are telehealth consultations (3)

○ 76-100% of my consultations are telehealth consultations (4)

○ I don’t know (5)

Q13.6 **Does your service plan to continue using telehealth (in some capacity) post COVID-19 when there are no restrictions on face-to-face consultations?**

○ Yes (1)

○ No (2)

○ I don’t know (3)

Display This Question:
If Does your service plan to continue using telehealth (in some capacity) post COVID-19 when there a... = No

Q13.7 **Why does your service not intend to continue using telehealth consultations post COVID-19?** (select all that apply)

- ▢ The patients didn’t like it (1)
- ▢ The patients couldn’t use the required technology to facilitate the conversation (2)
- ▢ Telehealth consultations have resulted in poorer clinical outcomes for patients (3)
- ▢ Telehealth consultations took longer than face-to-face consultations (4)
- ▢ Telehealth consultations are not appropriate for our patient groups (5)
- ▢ The staff didn’t have the skills necessary to conduct a telehealth appointment properly (6)
- ▢ The technology wasn’t reliable (7)
- ▢ The software wasn’t reliable (8)
- ▢ We didn’t get the same income for a telehealth consultation compared to a face-face consultation (9)
- ▢ The referrers stipulated that a face-to-face consultation was preferred (10)
- ▢ Other (please specify) (11) ____

Q13.8 **Do you think your service should continue using telehealth (in some capacity) post COVID-19, when there are no restrictions on face-to-face consultations?**

- ○ Yes (1)
- ○ No (2)
- ○ I don’t know (3)

Display This Question:

If Do you think your service should continue using telehealth (in some capacity) post COVID-19, when... = Yes

Q13.9 **Why do you think your service should continue using telehealth (in some capacity) post COVID-19?** (select all that apply)

- ▢ I would learn new skills (1)
- ▢ It is easier to arrange multidisciplinary team (MDT) consultations via telehealth (2)
- ▢ I can observe the patient in their own surroundings (3)
- ▢ I can see more patients per day (4)
- ▢ It allows flexible working patterns (5)
- ▢ It reduces my potential exposure to communicable diseases (6)
- ▢ It gives patients flexibility in how their consultations are conducted (7)
- ▢ It reduces the burden of treatment for some patients (8)
- ▢ The more we use telehealth consultations the more accepted they will be by patients and/or staff (9)
- ▢ Lower did not attend (DNA) rates (11)
- ▢ Other (please specify) (10) ____

Display This Question:
If Do you think your service should continue using telehealth (in some capacity) post COVID-19, when... = No

Q13.10 **Why don't you think your service should continue using telehealth (in some capacity) post COVID-19?** (select all that apply)

- ▢ Telehealth consultations take longer to conduct than a face-to-face consultation (1)
- ▢ Telehealth consultations are difficult to conduct (2)
- ▢ Telehealth consultations increase my stress levels (3)
- ▢ I don't feel I give my patients an optimum service via a telehealth consultation (4)
- ▢ Telehealth consultations reduce my physical activity levels during my working day (5)
- ▢ Telehealth consultations increase the amount of screen time I am exposed too (6)
- ▢ Other (please specify) (7) ____

Q13.13 **What benefit(s) to you, if any, have resulted from the introduction of telehealth consultations in your service?** (select all that apply)

- [ ] I have learned new skills (1)
- [ ] I think it is easier to arrange multidisciplinary team (MDT) consultations via telehealth (2)
- [ ] I can observe the patient in their own surroundings (3)
- [ ] I can see more patients per day (4)
- [ ] It allows flexible working patterns (5)
- [ ] It reduces my potential exposure to communicable diseases (6)
- [ ] I save travel time and costs (9)
- [ ] It is easier to network with colleagues and/or students (11)
- [ ] It optimises the time available for a consultation (13)
- [ ] Other (please specify) (7) ____
- [ ] ⊗I haven't identified any benefits to me from the introduction of telehealth consultations (8)

Q13.11 **What benefit(s) to patient groups, if any, have resulted from the introduction of telehealth consultations in your service?** (select all that apply)

- ▢ Not having to physically attend multiple appointments across the hospital or across different healthcare sectors. (1)
- ▢ A reduction in the cost to physically attend appointments e.g., petrol, parking, public transport costs (2)
- ▢ Not having to take time off work to attend appointments (3)
- ▢ Not having to ask a family member or carer to transport them to appointments (4)
- ▢ It reduces their potential exposure to communicable diseases (5)
- ▢ They feel more empowered to influence their health outcomes in their own environment (6)
- ▢ Patients who have disabilities/medical conditions which make telehealth consultations easier for them than face-to-face consultations (7)
- ▢ It improves the quality of care (11)
- ▢ It optimises the time available for a consultation (12)
- ▢ Ability for patients in rural/remote locations to access services (10)
- ▢ It allows the service to prioritise face-to-face consultations for essential and/or urgent consultations (13)
- ▢ Other (please specify) (8) ____
- ▢ ⊗I haven’t identified any benefits to patient groups from the introduction of telehealth consultations (9)

Q13.12 **What disadvantage(s) to** patient groups**, if any, have resulted from the introduction of telehealth consultations in your service?** (select all that apply)

▢ Having disabilities/medical conditions which makes telehealth consultations more difficult than face-to-face consultations e.g., visual and hearing impairments, patients with learning disabilities (1)

▢ Not having access to technology (2)

▢ Not having the necessary technology skills (3)

▢ Not wanting to use technology (4)

▢ I don’t feel my patients receive an optimum service via a telehealth consultation (8)

▢ There are issues with providing interpreter support during telehealth consultations (9)

▢ I found patients were unable to receive some treatments and/or to undergo assessment for specific dysfunctions (10)

▢ Telehealth consultations may have a higher potential to compromise the confidentiality of patient data in compliance with regulations and policies governing practice than face to face consultations (11)

▢ Other (please specify) (6) ____

▢ ⊗I haven’t identified any disadvantages to patient groups from the introduction of telehealth consultations (7)

Q13.14 **What disadvantage(s) to** you**, if any, have resulted from the introduction of telehealth consultations in your service?** (select all that apply)

- ☐ Telehealth consultations take longer to conduct than a face-to-face consultation (1)
- ☐ I find telehealth consultations difficult to conduct (2)
- ☐ Telehealth consultations increase my stress levels (3)
- ☐ Telehealth consultations are more tiring/draining (12)
- ☐ There is a loss of communication via body language in telehealth consultations (15)
- ☐ Telehealth consultations require increased concentration (16)
- ☐ Telehealth consultations contribute to my social isolation (11)
- ☐ I don't feel I give my patients an optimum service via a telehealth consultation (4)
- ☐ I don't want to change the way I conduct my patient consultations (5)
- ☐ Telehealth consultations reduce my physical activity levels during my working day (6)
- ☐ Telehealth consultations increase the amount of screen time I am exposed too (7)
- ☐ I have had to provide my own technology for telehealth consultations (8)
- ☐ Other (please specify) (9) ____
- ☐ ⊗I haven't identified any disadvantages to me from the introduction of telehealth consultations (10)

End of Block: Section 2 Telehealth use in your service (Clinician - has used telehealth)

Start of Block: Section 3 Telehealth consultation guidance in your service (clinician)

Q14.1 **Has your service implemented guidance on how telehealth consultations should be set-up?**

- ○ Yes (1)
- ○ No (2)
- ○ I don't know (3)

Skip To: End of Block If Has your service implemented guidance on how telehealth consultations should be set-up? = No

Display This Question:
If Has your service implemented guidance on how telehealth consultations should be set-up? = Yes

Q14.2 **Who produced the guidance?** (select all that apply)

- ☐ Your Professional body (1)
- ☐ Your NHS Trust (2)
- ☐ Your contractor (if you are not employed directly by the NHS) (3)
- ☐ The AHP service manager (4)
- ☐ Me, as the AHP manager (7)
- ☐ I don't know (5)
- ☐ Other (please specify) (6) ____

Q14.3 **Have you used the guidance to inform your telehealth consultations?**

- ○ Yes (1)
- ○ No (2)

Display This Question:
If Have you used the guidance to inform your telehealth consultations? = No

Q14.4 **Why haven't you used the guidance to inform your telehealth consultations?** (select all that apply)

- ☐ I don't think I need guidance for my telehealth consultations (1)
- ☐ I haven't read the guidance yet (2)
- ☐ I didn't understand the guidance (3)
- ☐ The guidance is unclear/ambiguous, where it is left to me to decide (4)
- ☐ The guidance lacks information or details on certain areas (for example, guidance on which patient groups are suitable for telehealth consultations, how to ensure minimal treatment delays for patients who have had an unsuccessful telehealth consultation, patient confidentiality and information governance or guidance on the use of technology to facilitate telehealth consultations) (5)
- ☐ Other (please specify) (6) ____

Display This Question:
If Have you used the guidance to inform your telehealth consultations? = Yes

Q14.5 **Do you think that your service guidance on telehealth consultations:**

	Yes (1)	No (2)	I don't know (3)
has areas of ambiguity, where it is left to you to decide (1)	○	○	○
lacks information or details on certain areas (for example, guidance on which patient groups are suitable for telehealth consultations, how to ensure minimal treatment delays for patients who have had an unsuccessful telehealth consultation, patient confidentiality and information governance or guidance on the use of technology to facilitate telehealth consultations) (2)	○	○	○

Display This Question:

If Do you think that your service guidance on telehealth consultations: [Yes] (Count) >= 1

Q14.6 **What area(s) of the guidance are ambiguous or lack information?** (select all that apply)

▢ Recommended consultation length (1)

▢ How to conduct an assessment without physical contact (2)

▢ Which patients are suitable for a telehealth consultation (3)

▢ Which treatment/interventions are appropriate for a telehealth consultation (4)

▢ How the clinician is protected from litigation should a telehealth consultation go wrong (5)

▢ How to deal with an emergency during a telehealth consultation (6)

▢ Training requirements for telehealth consultations (7)

▢ Other (please specify) (8) ____

Q14.7 **Does the guidance advise on:**

	Yes (1)	No (2)	I don't know (3)	Not applicable (4)
How to triage a referral for a telehealth consultation? (1)	○	○	○	○
The appropriate environment for staff to conduct a telehealth consultation? (2)	○	○	○	○
The appropriate environment for a patient to conduct a telehealth consultation? (3)	○	○	○	○
The appropriate technology for staff to conduct a telehealth consultation? (4)	○	○	○	○
The appropriate technology for a patient to conduct a telehealth consultation? (5)	○	○	○	○
The medical conditions which can be treated via a telehealth consultation? (6)	○	○	○	○
The type of consultation which can be conducted via a telehealth consultation (e.g. first assessment, follow up/review, issue of advice, issue of a piece of equipment or a medical device etc.)? (7)	○	○	○	○
The recommended consultation length required for each consultation type? (8)	○	○	○	○
Which patient groups might not be able to engage via a telehealth consultation? (9)	○	○	○	○
How to ensure patient groups who might not be able to engage via a telehealth consultation are identified and appointed appropriately ensuring they do not have to wait longer that another patient with the same clinical urgency? (10)	○	○	○	○
How to conduct a telehealth consultation with a patient who requires an interpreter? (11)	○	○	○	○
How to conduct a telehealth consultation with a patient who has a physical, mental, learning or communication disability? (12)	○	○	○	○
How to conduct a telehealth consultation with patients from different age groups? (13)	○	○	○	○

Q14.8 **Does the guidance advise on how patients with physical, mental, learning or communication disabilities or those who require an interpreter should be identified and triaged to ensure they are given an appropriate consultation to meet their needs?**

○ Yes, these patient groups are not offered a telehealth consultation only a face-to-face consultation (1)

○ Yes, we offer these patients a telehealth consultation first and if this is unsuccessful, they are then offered face-to face consultation (2)

○ No, the guidance does not offer advice on which consultation method these patients should receive (3)

○ I don't know (5)

○ Other (please specify) (4) ____

End of Block: Section 3 Telehealth consultation guidance in your service (clinician)

Start of Block: Section 4 Telehealth consultation training in your service (clinician)

Q15.1 **Have you been given training on the following?**

	Yes (1)	No, but I don't need training (2)	No, and I need training (3)
How to use the hardware required for conducting telehealth consultations? (1)	◯	◯	◯
How to use the software required for conducting telehealth consultations? (2)	◯	◯	◯
How to conduct a risk assessment prior to starting a telehealth consultation? (for example, assessing the patient's environment before commencing a telehealth consultation) (3)	◯	◯	◯
How to conduct a clinical assessment via a telehealth consultation? (4)	◯	◯	◯
How to provide a treatment intervention via a telehealth consultation? (5)	◯	◯	◯
How to ensure patient confidentially when conducting telehealth consultations? (6)	◯	◯	◯
How to get feedback from patients and their families about telehealth consultations? (7)	◯	◯	◯
How to deal with an emergency during a telehealth consultation (8)	◯	◯	◯

End of Block: Section 4 Telehealth consultation training in your service (clinician)

Start of Block: Section 5 Factors affecting telehealth consultations in your service (clinician)

Q16.1 **What barrier(s) involving you, if any, have affected your telehealth consultations?** (select all that apply)

▢ I haven’t received sufficient training (1)

▢ I don’t have access to the required technology (2)

▢ I don’t have sufficient technological skills (3)

▢ Lack of sufficient quality internet access (i.e., I have internet access, but the quality of the connection does not allow for a successful telehealth consultation) (4)

▢ I don’t have an appropriate environment to conduct telehealth consultation (5)

▢ My lack of engagement with telehealth consultations (6)

▢ Inadequate length of telehealth consultation (7)

▢ My requirement for reasonable adjustment (adjustments I require due to my disability, or physical or mental health condition(s)) (8)

▢ Other (please specify) (9) ____

▢ ⊗I haven’t identified any barrier(s), involving me, which have affected my telehealth consultations (10)

Q16.2 **What barrier(s) involving your patients, if any, have affected your telehealth consultations?** (select all that apply)

- ▢ Patient refusing to engage in a telehealth consultation (1)
- ▢ Lack of technology for patients (2)
- ▢ Lack of technological skills for patients (3)
- ▢ Lack of internet access for patients (4)
- ▢ Lack of sufficient quality internet access for patients (patients may have internet access but the quality of the connection does not allow for a successful telehealth consultation) (5)
- ▢ Lack of appropriate environment to conduct telehealth consultation for patients (6)
- ▢ Lack of engagement with telehealth consultations from patients (7)
- ▢ Lack of support from carer/family to facilitate telehealth consultation (13)
- ▢ Presenting condition/patient not appropriate for a telehealth consultation (8)
- ▢ The patient didn't think the telehealth consultation was a "real" consultation therefore wasn't prepared for the appointment (9)
- ▢ The patient didn't think the telehealth consultation was a "real" consultation therefore didn't attend (10)
- ▢ Other (please specify) (11) ____
- ▢ ⊗I haven't identified any barrier(s), involving my patients, which have affected my telehealth consultations (12)

End of Block: Section 5 Factors affecting telehealth consultations in your service (clinician)

Start of Block: Section 6 Use of Make Every Contact Count (MECC) in your service (clinician)

Q17.1 **Do you routinely have healthy behavior conversations in line with Make Every Contact Count (MECC) with your patients? e.g., conversations on weight loss, smoking and exercise.**

- ◯ Yes (1)
- ◯ No (2)

Display This Question:

If Do you routinely have healthy behavior conversations in line with Make Every Contact Count (MECC)... = Yes

Q17.2 **Does the use of telehealth consultations affect your MECC conversations?**

- ○ Yes, it makes them easier (1)
- ○ Yes, it makes them harder (2)
- ○ No (3)
- ○ I don't know (4)

End of Block: Section 6 Use of Make Every Contact Count (MECC) in your service (clinician)

Start of Block: Section 8 Anything else we should know

Q18.1 **Is there anything else that you think we should know about telehealth consultations in your service?**
(This is an optional question - please leave blank if you don't think there is anything else we should know)

____
____
____
____
____

End of Block: Section 8 Anything else we should know

Start of Block: Prize draw

Q103 Would you like to be entered in the prize draw to win a £100 retail voucher?

If you select yes we will ask for an email address to contact you should you win

- ○ Yes (1)
- ○ No (16)

Display This Question:
If Would you like to be entered in the prize draw to win a £100 retail voucher? If you select yes... = Yes

Q104 Please provide an email address to contact you should you win the prize draw

____

End of Block: Prize draw
